# Characterizing Olfactory Function in Children with Autism Spectrum Disorder and Children with Sensory Processing Dysfunction

**DOI:** 10.3390/brainsci10060362

**Published:** 2020-06-10

**Authors:** Julia R. Sweigert, Tanya St. John, Kristin Kawena Begay, Greg E. Davis, Jeffrey Munson, Eric Shankland, Annette Estes, Stephen R. Dager, Natalia M. Kleinhans

**Affiliations:** 1 Department of Radiology, University of Washington, Seattle, WA 98115, USA; shanklan@uw.edu (E.S.); srd@uw.edu (S.R.D.); 2 Department of Speech and Hearing Sciences, University of Washington, Seattle, WA 98115, USA; tstjohn@uw.edu (T.S.J.); estesa@uw.edu (A.E.); 3 Center on Human Development and Disability, University of Washington, Seattle, WA 98115, USA; 4 School of Education, University of Washington, Tacoma, WA 98402, USA; begayka@uw.edu; 5 Department of Otolaryngology, University of Washington, Seattle, WA 98115, USA; gedavis@uw.edu; 6 Department of Psychiatry and Behavioral Sciences, University of Washington, Seattle, WA 98115, USA; jeffmun@uw.edu; 7 Integrated Brain Imaging Center, University of Washington, Seattle, WA 98115, USA

**Keywords:** autism spectrum disorder, sensory processing, olfaction, smell detection, smell identification, PEA

## Abstract

Abnormalities in olfactory function have been identified in a number of neurological and psychiatric disorders, including Parkinson’s disease and schizophrenia. However, little is known about olfactory function in autism spectrum disorder (ASD). The present study aims to assess the olfactory profiles of children with ASD, compared to an age- and sex-matched comparison group of typically developing children and a second clinical control group consisting of non-ASD children with sensory processing dysfunction (SPD). Participants completed a battery of sensory and behavioral assessments including olfactory tasks (Sniffin’ Sticks Threshold Test and self-reported valence ratings for two target odorants (phenylethyl alcohol and vanillin) and the University of Pennsylvania Smell Identification Test), and an autism evaluation (Autism Diagnostic Observation Schedule-2). Children with ASD showed intact odor detection with reduced odor identification ability. Poor odor identification was significantly correlated with autism symptom severity. Children with SPD demonstrated reduced odor detection and identification ability. These findings provide evidence for differential patterns of smell processing among ASD and non-ASD neurodevelopmental disorders. Future studies are needed to determine whether the association of impaired olfaction and increased autism symptoms is due to shared etiology.

## 1. Introduction

Autism spectrum disorder (ASD) is defined by a combination of social communication deficits and the presence of restricted, repetitive behaviors [1]. Sensory dysfunction is recognized as part of the restricted, repetitive behaviors domain and is highly prevalent, with multiple sensory modalities affected in both children and adults with ASD [2]. The most commonly affected sensory modalities include auditory processing, tactile hypersensitivity, and increased sensory-seeking behaviors [3,4]. Importantly, symptoms of sensory dysfunction may further impair social functioning and may contribute to increased restrictive and repetitive behaviors [5]. 

Less is known about the olfactory domain in ASD than other sensory modalities. However, emerging evidence has shown a pattern of altered olfactory processing among some individuals with the disorder [6]. Efforts to characterize the olfactory profile of ASD remain inconclusive and prior studies are inconsistent with regard to the type of olfactory alteration found in ASD, variably identifying dysfunction in peripheral odor detection, secondary odor identification, or both [7,8,9,10,11,12,13,14,15].

Olfaction is strongly interconnected with the same social-emotional pathways implicated in ASD [16]. The primary olfactory cortex consists of the piriform cortex, entorhinal cortex, olfactory tubercle, and corticomedial nuclei of the amygdala [17], with secondary connections to the orbitofrontal cortex, hippocampus, hypothalamus, and other subregions of the amygdala [18,19]; this pathway allows for initial detection and interpretation of odorant stimuli, as well as secondary higher order processing, including associations with memory and emotion [20]. Among individuals with autism, structural and functional alterations have been identified in some of these regions, including the piriform cortex [12], the orbitofrontal cortex [21], and the amygdala [22,23]. Thus, studying olfaction may help also to increase understanding of social-emotional dysfunction in ASD. 

The present study aims to better characterize olfactory function in ASD, namely, odor detection threshold and odor identification, and to discover how these processes relate to ASD symptomology in a sample of children with ASD relative to two age- and sex-matched control groups: typically developing children with no history of developmental or psychiatric disorders and children with sensory processing dysfunction (SPD) but do not meet criteria for ASD. Children with SPD exhibit a range of sensory symptoms, including various hyper- and hyposensitivities as well as sensory seeking and sensory avoidant behaviors across sensory modalities. Although these sensory symptoms may be a commonality between ASD and SPD, children with SPD do not exhibit the same degree of social and communication impairments, which is a core diagnostic feature of ASD. Groups were based on direct assessment by expert research clinicians. This design will help elucidate whether certain patterns of olfactory dysfunction are unique to ASD or are present in a broader class of sensory-related neurodevelopmental disorders. Consistent with the majority of previous studies on olfaction in ASD [7,8,9,10,13], we predict that children with ASD will show impaired odor identification with normal odor detection threshold compared to the typically developing comparison group, reflecting alterations in secondary olfactory processing brain regions. In contrast, we hypothesize that children with SPD will show differences in odor detection consistent with hypersensitivity to stimulus detection, indicating dysfunction at the level of primary olfactory processing. We predict that individuals in all groups will demonstrate increased detection of odors they perceive to have greater positive valence. Because of the shared neural substrates between higher-level olfactory perception and social-emotional processing, we predict that an association between odor identification and social communication deficits will be observed specifically in the ASD group, and that those children with poorer odor identification performance will also show greater severity of social impairments.

## 2. Materials and Methods

### 2.1. Participants

**Figure 1:**
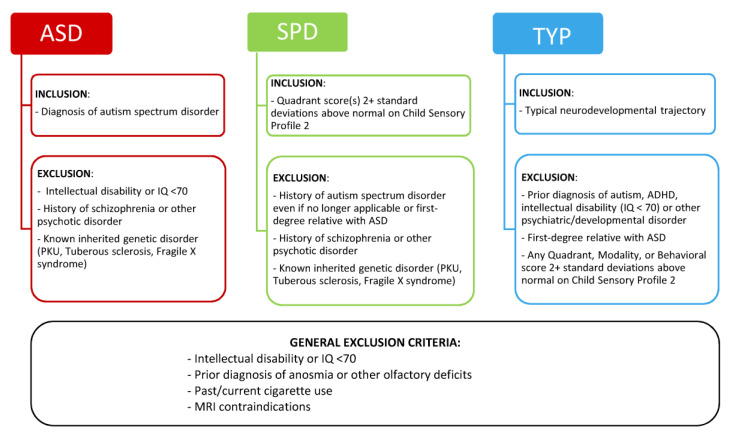
Eligibility and exclusion criteria for the three study groups. ASD = children with Autism Spectrum Disorder; SPD = children with demonstrated Sensory Processing Dysfunction; TYP = Typically Developing children.

Families with a child aged 7–13 years old were recruited from the Seattle metropolitan area via multiple resources, including flyers in community spaces, advertising on our laboratory website, postings in online forums for Seattle area parents, and a research registry at the University of Washington. During a semistructured telephone interview conducted with the parent or legal guardian, participants were screened for group assignment and study exclusion criteria. This study utilized three groups: children with an autism spectrum disorder (ASD), children with sensory processing dysfunction (SPD) including hypersensitivities and/or hyposensitivities, and typically developing children (TYP). The eligibility and exclusion criteria for each group are listed in Figure 1. After initial screening, a total of 151 participants (51 ASD, 50 SPD, and 50 TYP) were enrolled in the study.

Following a structured clinical evaluation to confirm group assignment, eighteen participants were disqualified; confirmatory testing procedures and reasons for disqualification are described in detail below. Participant characteristics for the final sample of 133 participants (43 ASD, 44 SPD, and 46 TYP) are reported in Table 1. The ASD group was predominantly male, which is consistent with well-documented differences in diagnostic rates of ASD between males and females [24]; recruitment efforts were made to ensure that the SPD and TYP groups had similar male–female ratios to reduce confounding effects of sex. Similar effort was made to ensure that all three groups were matched on age as well.

### 2.2. Procedures

The following study procedures were approved by the University of Washington Human Subjects Division Institutional Review Board (IRB #2148). Informed written consent was obtained from each participant’s parent or legal guardian. In addition, all participants provided verbal assent for each study procedure.

#### 2.2.1. Clinical Evaluation and Group Assignment

The preliminary group assignment was initially determined during the screening interview. Subsequently, each child underwent a clinical evaluation with an experienced licensed clinical psychologist to confirm group assignment. This evaluation consisted of cognitive testing, a comprehensive ASD diagnostic evaluation, assessment of sensory processing symptoms, and screening for comorbid psychiatric disorders. The Wechsler Abbreviated Scale of Intelligence (WASI) [25] was used to assess cognition and intellectual performance and consisted of two verbal tasks (Vocabulary and Similarities) and two visuospatial tasks (Block Design and Matrix Reasoning), yielding a Full-Scale IQ as well as Verbal IQ and Performance (non-verbal) IQ scores. The ASD evaluation included both the Autism Diagnostic Interview—Revised [26], a semistructured interview conducted over the telephone with a parent or legal guardian, and the Autism Diagnostic Observation Schedule, Version 2 (ADOS) [27], a standardized observational assessment of communication, social interaction and play skills. All participants completed Module 3 of the ADOS. A parent or legal guardian completed the Child Sensory Profile 2 [28], to rate the presence of various sensory-related behaviors and responses. Comorbid psychiatric disorders were identified using the Kiddie Schedule for Affective Disorders and Schizophrenia (KSADS), which assesses for key symptoms of various psychiatric disorders, including various anxiety disorders, mood disorders, attention/behavioral disorders, and psychotic disorders [29].

Final group inclusion criteria are displayed in Figure 1. All participants in the ASD group met DSM-5 criteria for ASD, based on the Diagnostic Statistic Manual of Mental Disorders, 5th ed. (DSM-5) [1]. All participants in the SPD group scored at least two standard deviations above normal on at least one Quadrant score (Seeking/Seeker, Avoiding/Avoider, Sensitivity/Sensor, and Registration/Bystander) on the Child Sensory Profile 2 and did not meet criteria for ASD. All participants in the TYP group scored in the normal range on the Child Sensory Profile 2, did not meet criteria for ASD, and did not meet criteria for any psychiatric disorder on the KSADS. Participants in the ASD and SPD groups were excluded for symptoms of schizophrenia and other psychotic disorders on the KSADS. However, comorbid mood, anxiety, and developmental disorders were not exclusionary for the ASD or SPD groups. High rates of comorbid psychiatric disorders have been reported in ASD [30]; to ensure that any observed differences between the ASD and SPD groups were not confounded by psychiatric comorbidities, the same KSADS exclusionary criteria were applied to both groups. Additionally, participants across all three groups were excluded for presence of an intellectual disability, defined as full-scale IQ < 70 on the WASI, or prior diagnosis of or overt symptoms consistent with anosmia.

Eighteen participants were disqualified following the clinical evaluation, per group exclusion criteria (Figure 1). Four participants (2 ASD and 2 SPD) were disqualified for IQ < 70. Four participants (4 ASD) were disqualified for not meeting research criteria for ASD at the time of clinical evaluation. Four participants (3 SPD and 1 TYP) were excluded based on Child Sensory Profile 2 scores. One participant (1 SPD) failed to show for study procedures. Two participants (2 ASD) were unable to complete study procedures due to receptive language deficits not detected during initial screening. Two participants (2 TYP) were excluded due to presence of symptoms consistent with attention deficit hyperactivity disorder identified with the KSADS. One participant (1 TYP) was excluded due to detection of clinically significant olfactory dysfunction during testing that had not been reported during initial screening.

#### 2.2.2. Olfactory Testing

Prior to olfactory testing, patients were assessed for active rhinitis, allergies, or other sources of nasal congestion. Patients were allowed to consume only water for thirty minutes prior to the start of olfactory testing. Olfactory tasks were conducted in a well-ventilated room with no other sources of odor present.

The odor detection threshold was determined by the Sniffin’ Sticks Threshold Test [31]. This task utilized a 16-step dilution series with forced choice, staircase procedure to systematically identify the lowest concentration of odorant detected by an individual. At the start of each test, participants were briefly presented with the target odorant at highest concentration to familiarize them with the target odor. Then, for each dilution step, blindfolded participants were asked to smell three pens and identify the pen with the target odor. Two of the pens were “blanks” containing only an unscented propylene glycol solvent; the third contained the target odorant diluted within propylene glycol. The pens were presented birhinally and in an alternating order (target-blank-blank, blank-target-blank, blank-blank-target, repeat) to avoid any learning effects. Participants completed the Threshold Test twice, once using an odorant of phenylethyl alcohol, a rose-like floral odorant (hereafter referred to as “Roses”), and once using a target odorant of pure vanillin, a vanilla-smelling compound (see Appendix A). These odors were selected because both compounds are pure olfactory stimulants, with minimal trigeminal effect [32,33]. The two tests were separated by a 20-minute washout period, during which no odors were present. The sequence of the odorant trials (Vanilla-first or Roses-first) was counterbalanced within each of the three groups. Due to the attentional demands of this task, research staff documented any noncompliant behaviors that might invalidate a participant’s results. 

Following each threshold test, participants were asked to rate the valence or “pleasantness” of the target odor, using a 7-point Likert Scale. A score of −3 indicated a very unpleasant odor, a score of +3 indicated a very pleasant odor, and a score of 0 indicated a neutral odor.

Odor identification was assessed using the University of Pennsylvania Smell Identification Test (UPSIT). The UPSIT is a 40-item “scratch and sniff” multiple choice test to assess how well participants can identify different odors [34]. Participants were asked to scratch the square microcapsule and smell the released odor, and then select from four choices listed on the page to identify the odor. The microcapsules contained supra-threshold concentrations of odor to distinguish odor identification performance from individual variability in odor detection threshold [35].

### 2.3. Statistical Analyses

All statistical analyses were completed using SPSS Version 18.0 [36]. A significance threshold of *α* = 0.05 was used for all analyses.

Descriptive statistics for each group and variable were calculated. Differences in age and IQ scores between the ASD, SPD, and TYP groups were analyzed using a one-way ANOVA. Significant between groups differences were followed by post hoc Fisher’s least significant difference tests to determine which specific groups differed.

Group differences in odor detection threshold, odor identification, and odor valence were analyzed with independent-sample *t*-tests. A paired-samples *t*-test was conducted to assess whether groups reported greater preference to either Roses or Vanilla. Based on the results of the between groups analysis, we conducted post hoc Pearson correlation analyses to further explore the relationship between odor detection threshold, odor valence, and odor identification within the ASD group to assess whether and how olfactory function varies by target odorant or perceived valence. 

Pearson correlation tests were conducted in the ASD and SPD groups to assess the relationship between odor identification (UPSIT) and autism severity (ADOS calibrated scores). Specifically, the ADOS Comparison calibrated severity score (“Total”) and the individual calibrated severity scores for the Social Affect and Restricted Repetitive Behaviors domains were used to indicate severity of ASD symptoms. 

## 3. Results

### 3.1. Olfactory Function by Group

Complete results from the Sniffin’ sticks threshold test with the Rose odorant were obtained for 38 of 43 participants in the ASD group (88%), 42 of 44 participants in the SPD group (95%), and all 46 participants in the TYP group (100%); incomplete data was due either to a participant’s unwillingness to compete task or poor compliance during the task that invalidated the results (e.g., participant repeatedly guessing Pen #3 before smelling all three pens, participant attempting to taste rather than smell the pens). Among the ASD group who completed the task, four participants (11%) scored in the anosmic range and 11 participants (29%) scored in the hyposmic range. Among the SPD participants who completed the task, four participants (10%) scored as anosmic and 17 participants (40%) scored in the hyposmic range. In the TYP group, no participants scored as anosmic while 15 participants (33%) scored in the hyposmic range. These categories are based on age-related normative values provided in the Sniffin’ Sticks Threshold Test manual [37].

Complete results from the Sniffin’ Sticks Threshold Test with the Vanilla odorant were obtained for 39 of 43 participants in the ASD group (91%), all 44 participants in the SPD group (100%), and all 46 participants in the TYP group (100%). Published normative data is not available for the Vanilla odorant, so we are not able to report on Vanilla anosmia and hyposmia rates in the present sample.

Total scores on the UPSIT were obtained for 40 out of 43 ASD participants (93%), 41 out of 44 SPD participants (93%), and all 46 participants in the TYP group (100%). Of the 40 ASD participants with complete data, six participants (15%) scored in the anosmic range and 16 participants (40%) scored in the microsmic range. Among the 44 SPD participants with UPSIT data, four participants (9%) scored in the anosmic range and 15 participants (37%) scored in the microsmic range. No participants in the TYP group scored in the anosmic range, while 17 out of 46 TYP participants (37%) scored in the microsmic range. These categories are based on age- and gender-normative values provided in the UPSIT instruction manual [35]. 

Valence data was collected for a subset of participants in each group, as this measure was added for secondary analysis after data collection had begun. Roses valence data was provided by 26 out of 43 ASD participants (60%), 31 out of 44 SPD participants (70%), and 33 of 46 TYP participants (72%). Vanillin valence data was collected for 25 out of 43 ASD participants (58%), 31 out of 44 SPD participants (70%), and 32 out of 46 TYP participants (70%). A paired samples t-test reveals that the Vanilla odorant was rated as significantly more pleasant than the Roses odorant by the TYP group (M_Rose_ = 1.38, SD_Rose_ = 1.31, M_Vanilla_ = 2.00, SD_Vanilla_ = 1.16, t (31) = −2.552, *p* = 0.016) and the SPD group (M_Rose_ = 0.60, SD_Rose_ = 1.99, M_Vanilla_ = 1.80, SD_Vanilla_ = 1.61, t (29) = −2.485, *p* = 0.019). There was no significant difference in pleasantness ratings between the two odorants in the ASD group (M_Rose_ = 1.44, SD_Rose_ = 1.39, M_Vanilla_ = 2.08, SD_Vanilla_ = 1.55, t (24) = −1.539, *p* = 0.137).

### 3.2. Olfactory Function between Groups

Table 2 contains the results for each olfactory measure by group. Full results for between-groups comparisons of odor detection threshold, odor identification, and odor valence ratings are shown in Table 3.

**Figure 2:**
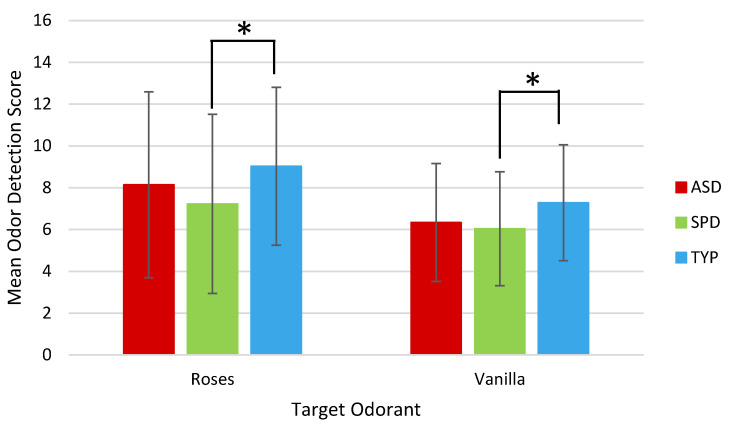
Mean odor detection threshold scores by group, as measured by Sniffin’ Sticks Odor Detection Threshold Test for target odors of Roses and Vanilla. Higher threshold scores represent detection at lower odorant concentration. Error bars indicate standard deviation. (*) denotes significant at *p* < 0.05. ASD = children with Autism Spectrum Disorder; SPD = children with demonstrated Sensory Processing Dysfunction; TYP = Typically Developing children.

Participants in the ASD showed no significant difference in odor detection threshold scores, relative to the TYP group, for both the Roses odorant (*p* = 0.33) and the Vanilla odorant (*p* = 0.126). In contrast, the SPD group scored significantly lower on the odor detection threshold test for both Roses (*p* = 0.039) and Vanilla odorants (*p* = 0.035), compared to the TYP group (Figure 2). A comparison of the ASD and SPD groups revealed no significant between groups difference in odor detection threshold for either odorant.

**Figure 3:**
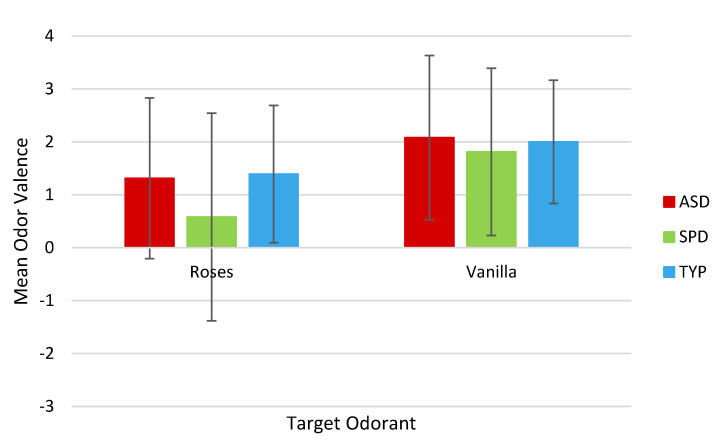
Mean odor valence scores by group, based on 7-point Likert Scale. −3 = Very Unpleasant, 0 = Neutral, +3 = Very Pleasant. No significant differences in odor valence were observed between the three groups at *α* = 0.05. ASD = children with Autism Spectrum Disorder; SPD = children with demonstrated Sensory Processing Dysfunction; TYP = Typically Developing children.

The ASD group did not differ from the TYP group in perceived valence of the Roses odorant (*p* = 0.82) or Vanilla odorant (*p* = 0.83). The SPD group rated the Roses odorant as less pleasant than the TYP group, but this result was not statistically significant (*p* = 0.054). For the Vanilla odorant, there was no difference in pleasantness rating between the SPD and TYP groups (*p* = 0.581) (Figure 3). No difference in valence rating was observed between the ASD and SPD groups for either odorant.

**Figure 4:**
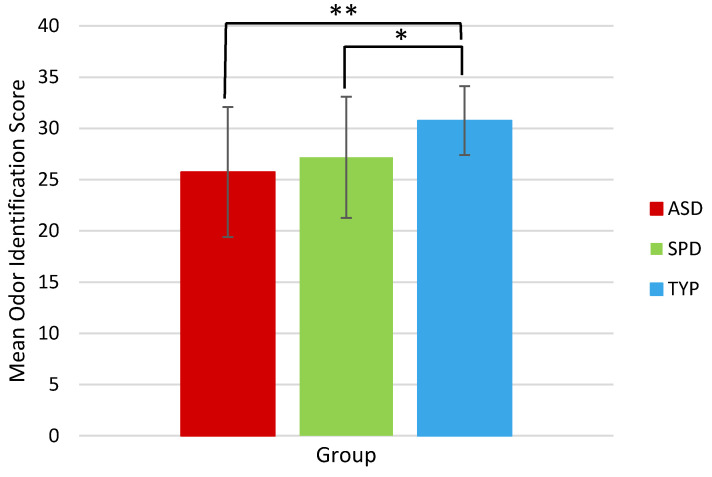
Mean odor identification scores by group, as measured by University of Pennsylvania Smell Identification Test (UPSIT). Error bars indicate standard deviation. (*) denotes significant at *p* < 0.01 (**) denotes significance at *p* < 0.001. ASD = children with Autism Spectrum Disorder; SPD = children with demonstrated Sensory Processing Dysfunction; TYP = Typically Developing children.

On the UPSIT, significantly reduced odor identification scores were observed in both the ASD group (*p* < 0.001) and SPD group (*p* = 0.001), compared to the TYP group (Figure 4). Direct comparison of ASD and SPD groups showed no significant difference in odor identification (*p* = 0.29). 

### 3.3. Olfactory Function Correlations

Table 4 contains results for the correlation analyses between the different olfactory metrics. 

Across all groups, Roses detection threshold was not significantly correlated with Roses valence. Vanillin detection threshold was significantly correlated with Vanilla valence only in the ASD group (*r* (21) = −0.539, *p* = 0.008), with no such correlation observed in the SPD or TYP groups.

**Figure 5:**
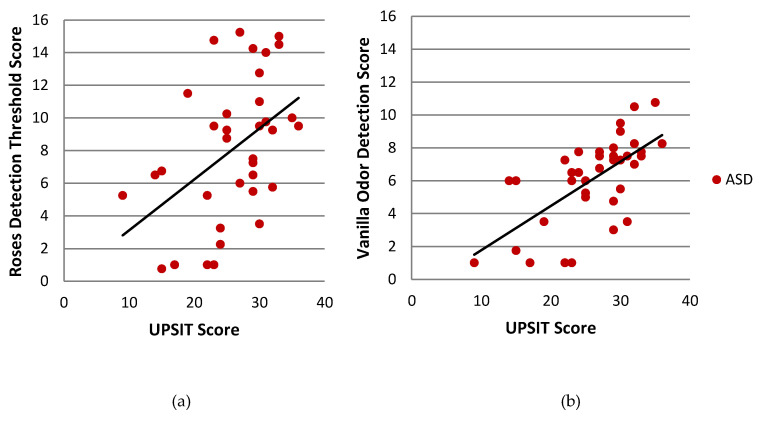
Correlation between smell identification (UPSIT Score) and Sniffin’ Sticks Threshold Test in the ASD group. Sniffin’ Sticks odor detection threshold scores are displayed for the Roses odor (**a**) and Vanilla odor (**b**). Each point represents one individual. UPSIT = University of Pennsylvania Smell Identification Test; ASD = children with Autism Spectrum Disorder.

Odor identification scores were significantly correlated with odor detection scores for Vanilla (*r* (35) = 0.649, *p* < 0.001)) and Roses (*r* (34) = 0.463, *p* = 0.004) in the ASD group (Figure 5). This relationship was not observed in the TYP group (data not shown). In the SPD group, odor identification was correlated only with Roses odor detection (*r* (37) = 0.344, *p* = 0.032), but not Vanilla odor detection (data not shown).

**Figure 6:**
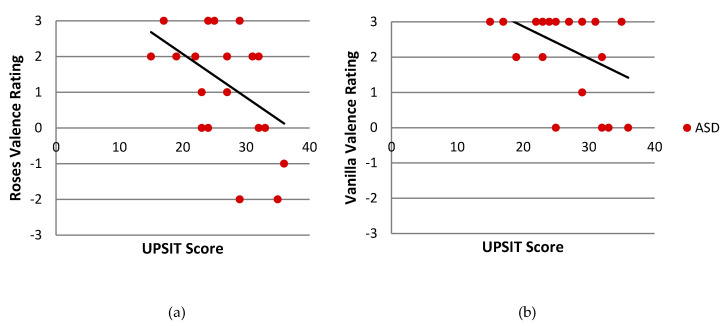
Within groups correlation between smell identification (UPSIT Score) and odor valence in the ASD group. Odor valence is displayed for the Roses odor (**a**) and Vanilla odor (**b**). Each point represents one individual. UPSIT = University of Pennsylvania Smell Identification Test; ASD = children with Autism Spectrum Disorder.

Similarly, in the ASD group, odor identification was correlated with reported valence of both Roses (*r* (23) = −0.422, *p* = 0.036) and Vanilla (*r* (22) = −0.425, *p* = 0.038) target odorants (Figure 6). The TYP group also showed a significant correlation between Roses valence and odor identification (*r* (31) = −0.361, *p* = 0.039). No significant correlations were observed in the SPD group (data not shown).

### 3.4. Odor Identification and ASD Severity

Full results for the correlation analyses between UPSIT scores and severity of ASD-related behaviors are available in Table 5. 

**Figure 7:**
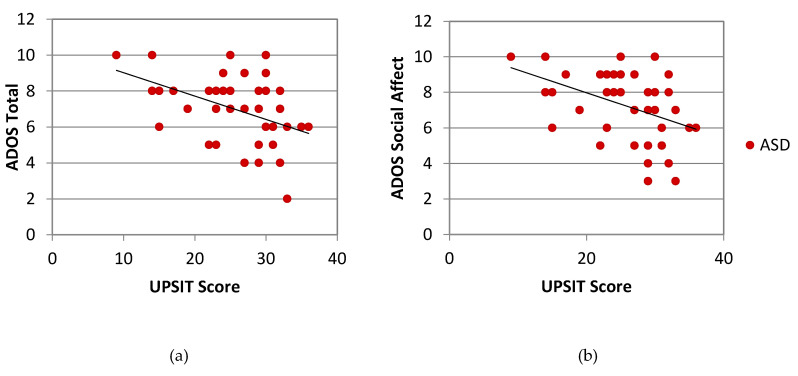
Within groups correlation between smell identification (UPSIT score) and autism severity in the ASD group. Autism severity is displayed separately as ADOS Total calibrated severity score (**a**) and Social Affect calibrated severity score (**b**). Each point represents one individual. UPSIT = University of Pennsylvania Smell Identification Test; ADOS = Autism Diagnostic Observation Schedule; ASD = children with Autism Spectrum Disorder.

Within the ASD group, UPSIT scores showed a strong negative correlation with ADOS Total Score (*r* (38) = −0.431, *p* = 0.005) and Social Affect (*r* (38) = −0.424, *p* = 0.006) (Figure 7), but not with Restrictive/Repetitive Behavior. Within the SPD group, UPSIT scores were not significantly correlated with ADOS Total, Social Affect, or Restricted/Repetitive Behavior (data not shown).

## 4. Discussion

This study investigated differences in olfactory function between children with ASD, SPD, and typical development. As predicted, we observed altered olfactory function in both the ASD and SPD groups. Relative to typically developing peers, children with ASD demonstrated impairments in odor identification but not odor detection. In contrast, children with SPD showed impairments in both odor detection threshold and odor identification compared to TYP children. Within the ASD group, differences in odor identification were associated with overall ASD severity and symptoms of social impairment. We did not see the same relationship among children with SPD.

Children with SPD were a particularly salient clinical control group because they present with some of the sensory hypo- and hypersensitivities also seen in ASD, but not the social communication deficits seen in ASD. This allowed for increased precisions in characterizing olfactory dysfunction specific to ASD versus olfactory dysfunction common to neurodevelopmental disorders generally. 

Our finding of olfactory dysfunction in both clinical groups is consistent with studies of olfaction in other disorders. Olfactory involvement has been well-documented in various neurological and psychiatric disorders, including Parkinson’s disease, schizophrenia, and depression [38,39,40]. Our results suggest that there appear to be distinct differences between children with ASD and children with SPD in how olfaction is impaired.

This study adds to the limited but growing body of literature on olfactory function in ASD, much of which still remains inconclusive. The present results are consistent with a subset of prior studies [7,8,9,10,13] that also found individuals with ASD to be impaired in their ability to identify odors with intact odor detection, suggesting ASD-related alterations at the level of secondary cortical processing. This is in contrast to other studies [11,12,14] that posit the higher-level deficits may be driven in part by impairments at the level of primary olfactory detection. Notably, 75% of the studies that reported impairments in odor detection [11,14,41] used samples of children with ASD, whereas six of the seven studies reporting normal [9,10,13,42] or enhanced [15] smell detection utilized adult samples, indicating that there may be developmental differences in olfactory dysfunction among individuals with ASD, in addition to well-known symptom heterogeneity. Furthermore, the methodological advances employed by Muratori et al. [11] and Kumazaki et al. [41] may be more sensitive to mild olfactory dysfunction than traditional odor testing; these new methodologies are worth exploring further. Another extant theory in the field proposes that individuals with ASD actually have higher sensitivity to odor and taste stimuli [6,15,43]. Our findings, which are consistent with the majority of studies, suggest that this sensitivity is not actually a result of enhanced detection within primary olfactory cortices. Instead, there may be differences in how this chemosensory information is processed and interpreted within the brain, and how people with ASD respond to olfactory information; task-based neuroimaging studies may help to further elucidate the neural mechanisms underlying these differences.

This suggests that the olfactory dysfunction occurring in individuals with SPD may arise from differences in how stimuli are sensed at the peripheral level, in addition to abnormalities in central perceptual processing. To our knowledge, this is the first study to assess the olfactory profiles of children with SPD. Prior studies have observed heightened odor detection among children with ADHD, see, for example, in [44,45], but our results demonstrate that children with SPD may have relatively more difficulty detecting odor stimuli compared to other children. This unexpected finding may be in part due to differences in diagnostic criteria, where our group inclusion depended on general hypo- and hypersensitivities across sensory domains, rather than attention symptoms. It is unlikely that this difference in odor identification is driven by impaired odor detection, as the UPSIT presents odors at suprathreshold concentrations [35] and is thus designed to remove any confounding effects relating to differences in odor detection. Rather, olfactory function in these children is compounded by dysfunction at multiple steps along the pathway, which differs from the pattern we observed in children with ASD.

This differential pattern of olfactory dysfunction could point to separate mechanisms underlying the symptoms associated with these two neurodevelopmental disorders. Previous research has identified relationships between olfactory function and the volume of associated brain regions, including odor detection associated with olfactory bulb size [45] and odor identification/discrimination associated with secondary olfactory region volumes [46,47]. An investigation that compares differential brain activity during olfactory tasks in these pediatric populations may help to pinpoint the neural alterations responsible for the somewhat unique olfactory profiles of these two groups.

We observed that among children with ASD, those with greater odor identification dysfunction also showed more severe ASD symptomatology, in particular, social deficits. This finding points to a possible shared mechanism underlying both sensory and social symptoms of ASD. Of important consideration, lesion studies and neuroimaging have identified the orbitofrontal cortex as a critical area for conscious odor perception and identification [48,49,50], and a subpopulation of neurons in the orbitofrontal cortex has also been identified in face recognition and social reinforcement [46]. We know that facial recognition and social motivation processes are altered in people with ASD and have been associated with altered patterns of brain activity [51,52,53]. Interestingly, odor valence has also been linked to neural activity in the orbitofrontal cortex [54,55,56]. That we also observed a relationship between odor identification and valence lends further support to the implication of the orbitofrontal cortex in both social and olfactory symptoms of ASD.

While children with SPD demonstrate impaired detection of both the Vanilla and Roses odorants and children with ASD generally appear to have intact peripheral detection based on the present findings, there appeared to be some individual variation according to the odorant type. Vanillin odor detection was associated with odor identification only in the ASD group, whereas Roses odor detection was associated with identification in both the ASD and SPD groups. As mentioned before, because the UPSIT is designed to reduce the influence of individual variability in odor detection threshold by using suprathreshold concentrations, this association does not necessarily indicate that reduced odor detection is driving odor identification impairments. Instead, this finding may actually point to a subset of individuals with ASD who do in fact show dual impairments at both the detection and identification stages of olfactory processing. Two recent studies [11,12] have also observed impairments at both levels. This relationship was the strongest for detection of the Vanilla odorant, which suggests that there may be some receptor selectivity in those individuals with peripheral detection impairments.

Ultimately, these findings provide further evidence that the olfactory system appears to have unique properties that make it more susceptible to dysfunction when neuropsychiatric pathologies are present. As we learn more about these disorders, we may come to a better understanding of how the olfactory system is impacted and also how we may use olfactory testing to detect the earliest stages of disorders such as ASD. 

### Limitations

The olfactory testing battery assessed three aspects of the olfactory pathway: odor detection threshold, odor identification, and odor valence. This battery did not include other dimensions, such as odor discrimination (the ability to differentiate between two different odors), odor awareness [57], and odor memory. Additionally, we focused on odor detection threshold and subjective odor valence of only two odors, both of which are generally recognized as pleasant odors (floral and vanilla). Investigating these added metrics and utilizing a greater selection of odorants in the battery, including a subset of odors with negative and/or neutral valence, may allow further specification of the olfaction profiles of ASD and SPD. Furthermore, although all olfactory testing was conducted at a single site, data was not collected on temperature and humidity in the rooms where the olfactory testing was conducted; because temperature and humidity is known to influence the volatility of odorant molecules, it is possible that variations in these environmental conditions may have introduced error into our measurements.

Participants in all three groups were screened and excluded based on IQ < 70 to eliminate severe cognitive impairments as a possible confounding variable. This was necessary because secondary processing of olfactory cues rely on various cognitive processes that might inherently be reduced in children with intellectual disability. Unfortunately, this selection criterion does limit the generalizability of these findings, which may not be representative of individuals with more severe ASD or SPD and comorbid intellectual disability.

Due to high rates of comorbid psychiatric disorders in ASD, including affective disorders, anxiety disorders, and attentional disorders [30], we chose to include children with these disorders in our ASD group, and for matching purposes, in the SPD group as well. Olfactory alterations have been observed among people with depression [40], so it is possible that depression among some participants may contribute in part to the present findings of impaired olfactory function. To more specifically assess how olfaction varies in this pediatric population, future studies would do well to compare olfactory performance in children with ASD and comorbid depression versus ASD without comorbid depression.

The attentional demands of the Sniffin’ Sticks Threshold Test can make it a challenging task to complete for some children with ASD, SPD, and/or attention deficit hyperactivity disorder. Poor tolerance of this task may result in lower odor detection threshold scores, and thus it is possible that some of the between groups effects may be overestimated. To reduce this bias, we did eliminate a subset of Sniffin’ Sticks Threshold Test scores from the reported analyses due to validity concerns (discussed above in Section 2.2.2 Olfactory Testing) to ensure that the data used in the analyses represented true variability in odor detection thresholds, rather than compliance issues. However, future studies could utilize alternative testing methods that enhance attention to the task and further reduce attentional biases.

Last, because Sensory Processing Disorder has not yet been added to the DSM-5, we operationalized the criteria for this group based on Child Sensory Profile 2 scores. It is important to note that this group included a variety of sensory alterations; children with sensory seeking and/or sensory avoidant behavior, as well as children with hyper- and hyposensitivities across sensory modalities. This broad spectrum of sensory symptomatology has the advantage of encompassing the full range of sensory subtypes seen in children with ASD [58]. However, this heterogeneity may mask some of the more nuanced differences among individual children in this population. Future studies may benefit from utilizing larger study groups matched by type(s) of sensory processing alteration. This will extend our findings to assess how olfactory function may differ in relation to different sensory profiles.

## 5. Conclusions

To our knowledge, this study is the first to directly measure olfactory function in both children with ASD and children with SPD. Both the ASD group and the SPD group demonstrated impaired odor identification, which is consistent with observations among other neuropsychiatric disorders. Additionally, the SPD group also showed reduced odor detection, whereas children with ASD appeared to have intact odor detection. These findings suggest that while olfaction is disrupted in both neurodevelopmental disorders, the primary deficit likely occurs through different mechanisms and at different levels of the olfactory pathway. Furthermore, the association between odor identification deficits and severity of social impairments within the ASD group provides further evidence for involvement of the orbitofrontal cortex in these shared aspects of ASD symptomatology. This idea should be examined more closely in the future using neuroimaging paradigms that tap into both olfactory perception and socioemotional networks. Overall, these findings suggest that certain characteristics of the olfactory system may pose a particular vulnerability to neural alterations and/or serve as a proxy indicator for impairments of other social-emotional processes.

## Figures and Tables

**Table 1:** Participant characteristics.

	ASD (*n* = 43)		SPD (*n* = 44)		TYP (*n* = 46)		F	*p*-Value
	Mean	(SD)	Mean	(SD)	Mean	(SD)		
Gender (Male: Female)	39:4		38:6		39:7			
Age (yrs.)	10.21	(1.63)	9.98	(1.62)	10.32	(1.44)	0.54	0.59
Wechsler Abbreviated Scales of Intelligence								
Full-Scale IQ	112.33	(17.90)	115.64	(18.12)	119.35	(11.06)	2.16	0.12
Performance IQ	114.91	(14.33)	113.02	(16.80)	115.28	(13.12)	0.297	0.74
Verbal IQ	107.33	(21.21)	114.75	(18.07)	119.11	(11.19)	5.29	0.006 ^a,c^
Autism Diagnostic Observation Schedule ^d^								
Social Affect	7.37	(1.94)	3.73	(2.56)	1.98	(1.22)	85.54	<0.001 ^a,b,c^
Restricted/Repetitive Behav.	6.67	(2.33)	3.50	(2.26)	1.80	(1.80)	59.31	<0.001 ^a,b,c^
Comparison Score	7.12	(1.95)	3.16	(2.22)	1.43	(0.81)	121.08	<0.001 ^a,b,c^
Child Sensory Profile 2								
Avoidant	55.53	(14.50)	58.57	(14.82)	26.93	(7.43)	86.18	<0.001 ^a,b^
Seeking	42.02	(16.47)	51.05	(14.62)	22.74	(7.32)	53.38	<0.001 ^a,b,c^
Sensitive	51.47	(13.10)	52.18	(13.56)	22.28	(6.77)	97.51	<0.001 ^a,b^
Bystander	52.14	(15.56)	54.27	(16.80)	25.02	(8.54)	61.03	<0.001 ^a,b^
Auditory	24.16	(6.64)	25.57	(6.69)	11.85	(4.01)	74.39	<0.001 ^a,b^
Body Position	17.28	(7.76)	19.11	(9.21)	8.26	(3.26)	29.80	<0.001 ^a,b^
Movement	15.65	(7.42)	18.27	(6.79)	8.13	(3.32)	33.99	<0.001 ^a,b^
Oral	25.58	(10.13)	26.70	(10.38)	11.39	(4.68)	43.08	<0.001 ^a,b^
Touch	20.77	(9.81)	25.73	(9.63)	10.91	(4.10)	37.96	<0.001 ^a,b,c^
Visual	14.09	(5.22)	14.80	(4.80)	10.00	(3.29)	15.03	<0.001 ^a,b^
Attention Behavior	26.63	(6.59)	26.73	(7.29)	11.50	(4.58)	89.26	<0.001 ^a,b^
Conduct Behavior	22.81	(7.97)	25.52	(7.30)	11.78	(4.22)	54.03	<0.001 ^a,b^
Social Emotional Behavior	41.44	(11.43)	43.20	(12.84)	19.43	(5.91)	72.78	<0.001 ^a,b^

^a^ Sig. Diff. between TYP and ASD; ^b^ Sig. Diff. between TYP and SPD; ^c^ Sig. Diff. between ASD and SPD; ^d^ All Autism Diagnostic Observation Schedule data are presented as calibrated severity scores; ASD = children with Autism Spectrum Disorder; SPD = children with Sensory Processing Dysfunction; TYP = Typically Developing children.

**Table 2:** Olfactory function by group.

	ASD		SPD		TYP	
	*s*	(SD)	Mean	(SD)	Mean	(SD)
Sniffin’ Sticks Threshold Test						
Roses	8.14	4.45	7.23	4.29	9.03	3.78
Vanilla	6.34	2.82	6.04	2.72	7.28	2.78
Valence Rating						
Roses	1.31	1.52	0.58	1.96	1.39	1.30
Vanilla	2.08	1.55	1.81	1.58	2.00	1.16
UPSIT	25.73	6.35	27.17	5.91	30.76	3.36

ASD = children with Autism Spectrum Disorder; SPD = children with demonstrated Sensory Processing Dysfunction; TYP = Typically Developing children; UPSIT = University of Pennsylvania Smell Identification Test.

**Table 3:** Olfactory function between groups.

	ASD v. TYP			SPD v. TYP			ASD v. SPD		
	df	*t*	*p*-Value	df	*t*	*p*-Value	Df	*t*	*p*-Value
Sniffin’ Sticks Threshold Test									
Roses	82	0.991	0.33	86	2.095	0.039 *	78	0.933	0.35
Vanilla	83	1.547	0.126	88	2.146	0.035 *	81	0.495	0.62
Valence Rating									
Roses	57	0.235	0.82	62	1.967	0.054	55	1.54	0.13
Vanilla	55	−0.222	0.83	61	.555	0.581	54	0.649	0.52
UPSIT	84	4.683	<0.001 ***	85	3.532	0.001 **	79	−1.062	0.29

* denotes significant at *p* < 0.05; ** denotes significant at *p* < 0.01; *** denotes significant at *p* < 0.001; ASD = children with Autism Spectrum Disorder; SPD = children with demonstrated Sensory Processing Dysfunction; TYP = Typically Developing children; UPSIT = University of Pennsylvania Smell Identification Test.

**Table 4:** Correlations between olfactory metrics.

	ASD Group			SPD Group			TYP Group		
	DF	*r*	*p*-Value	DF	*R*	*p*-Value	DF	*r*	*p*-Value
Sniffin’ Sticks—Roses									
Valence Rating—Roses	21	−0.043	0.85	27	−0.116	0.55	31	0.115	0.53
Sniffin’ Sticks—Vanilla									
Valence Rating—Vanilla	21	−0.539	0.008 **	29	.053	0.78	30	−0.048	0.793
UPSIT									
Sniffin’ Sticks—Roses	34	0.463	0.004 **	37	0.344	0.032 *	44	0.089	0.56
Sniffin’ Sticks—Vanilla	35	0.649	0.000 ***	39	0.217	0.17	44	−0.196	0.19
UPSIT									
Valence Rating—Roses	23	−0.422	0.036 *	27	0.271	0.16	31	−0.361	0.039 *
Valence Rating—Vanilla	22	−0.425	0.038 *	27	0.107	0.58	30	0.050	0.79

* denotes significant at *p* < 0.05; ** denotes significant at *p* < 0.01; *** denotes significant at *p* < 0.001; ASD = children with Autism Spectrum Disorder; SPD = children with demonstrated Sensory Processing Dysfunction; TYP = Typically Developing children; UPSIT = University of Pennsylvania Smell Identification Test.

**Table 5:** Correlations between UPSIT and ADOS Scores ^a^.

	ASD Group			SPD Group		
	DF	*r*-Value	*p*-Value	DF	*r*-Value	*p*-Value
ADOS Total	38	−0.431	0.005 **	42	−0.230	0.13
ADOS Social Affect	38	−0.424	0.006 **	42	−0.222	0.15
ADOS Restricted/Repetitive Behavior	38	−0.171	0.29	42	−0.044	0.78

* denotes significant at *p* < 0.05; ** denotes significant at *p* < 0.01; *** denotes significant at *p* < 0.001; ^a^ All Autism Diagnostic Observation Schedule data were analyzed using calibrated severity scores; ASD = children with Autism Spectrum Disorder; SPD = children with Sensory Processing Dysfunction; UPSIT = University of Pennsylvania Smell Identification Test; ADOS = Autism Diagnostic Observation Schedule.

## Appendix A

Dilutions of ethylvanillin [1, Sigma-Aldrich: W246409] were prepared in propylene glycol [2, Sigma-Aldrich: P4347]. Specifically, a 4% [*w*/*w*] stock solution was prepared by dissolving 0.249 g of 1 in 6 mL [6.216 g, density 1.036 g∙ms/mL] of 2, noting complete dissolution. Fifteen additional bottles were filled with 4 mL of 2. Dilutions were prepared taking 2 mL of stock solution to add to a 2nd bottle bringing its volume to 6 mL with vigorous mixing. Subsequent dilutions were similarly prepared. The 4 mL of each dilution was added to numbered, red pens for experiments. An additional set of 16 of green and blue pens were filled with propylene glycol, alone, for control.
